# The Vitamin E Derivative Garcinoic Acid Suppresses NLRP3 Inflammasome Activation and Pyroptosis in Murine Macrophages

**DOI:** 10.1007/s10753-025-02269-6

**Published:** 2025-02-21

**Authors:** Lisa Börmel, Anja R. Geisler, Yvonne Hupfer, Sijia Liao, Tina Schubert, Stefan Kluge, Stefan Lorkowski, Maria Wallert

**Affiliations:** Institute of Nutritional Science, Friedrich Schiller University, 07743 Jena, Germany

**Keywords:** NLRP3 inflammasome, Pyroptosis, Macrophages, Vitamin E, Garcinoic acid

## Abstract

Excessive inflammation in cells are a common cause of inflammation-related diseases such as cardiometabolic diseases. The cellular multiprotein complex nucleotide-binding domain and leucine-rich repeat pyrin domain 3 (NLRP3) inflammasome is a cellular key modulator of inflammatory processes. In addition to classic medications, phytochemicals are known for their anti-inflammatory potential. In African folk medicine the seeds of *Garcinia kola* are used to support the treatment of inflammatory diseases. Of particular interest is the phytochemical garcinoic acid (GA, *trans*-13′-carboxy-δ-tocotrienol), which is isolated from the *Garcinia kola* seeds. This derivative and potential metabolite of the vitamin E congener δ-tocotrienol (T3) shows anti-inflammatory properties in vitro. However, the underlying mechanisms are largely unknown. To get better insights into the molecular mode of action, murine J774A.1 macrophages were stimulated with lipopolysaccharides (LPS) only or in combination with adenosine triphosphate (ATP), which led to canonical activation of the NLRP3 inflammasome and subsequent pyroptosis. A combined treatment with GA resulted in significantly reduced stimulation of the transcription factor nuclear factor 'ĸ-light-chain-enhancer' of activated B-cells (NF-ĸB), decreased expression of inflammasome-related genes and marked downregulation of autoproteolytic cleavage of caspase-1 (Casp-1). Consequently, GA had an inhibitory effect on pyroptosis. The results have been validated using the well-known NLRP3 inflammasome inhibitor MCC950. In conclusion, GA was shown to have relevant effects on the regulation of the NLRP3 inflammasome and pyroptosis in vitro. Our study provides new mechanistic insights into the anti-inflammatory mode of action of GA and highlights its relevance as a potential phytochemical drug for the treatment of inflammation.

**Supplementary Information:**

The online version contains supplementary material available at 10.1007/s10753-025-02269-6.

## Introduction

Noncommunicable diseases (NCDs) are currently the world's number one cause of death. Cardiovascular diseases, cancers, chronic respiratory diseases and diabetes are the four major diseases responsible for about 80% of premature NCD-related deaths. The development of these NCDs are usually due to a combination of genetic, physiological, environmental and lifestyle factors. In addition, the people affected, may have an elevated inflammatory status [1, 2]. Inflammation is a crucial innate immune defense mechanism in response to harmful stimuli. However, if this endogenous reaction is uncontrolled, it becomes the basis for NCDs, a condition known as chronic inflammation [3].

Over the past two decades, the nucleotide-binding domain and leucine-rich repeat pyrin domain 3 (NLRP3) inflammasome, a cytosolic multiprotein complex, has been extensively studied for its critical role in regulating the cellular inflammatory response. The most extensively studied inflammasome structure to date consists of a sensor protein called NLRP3, an adaptor protein called apoptosis-associated speck-like protein containing a caspase-recruitment domain (ASC), and an effector protein called caspase-1 (Casp-1) [4, 5].

**Fig. 1:**
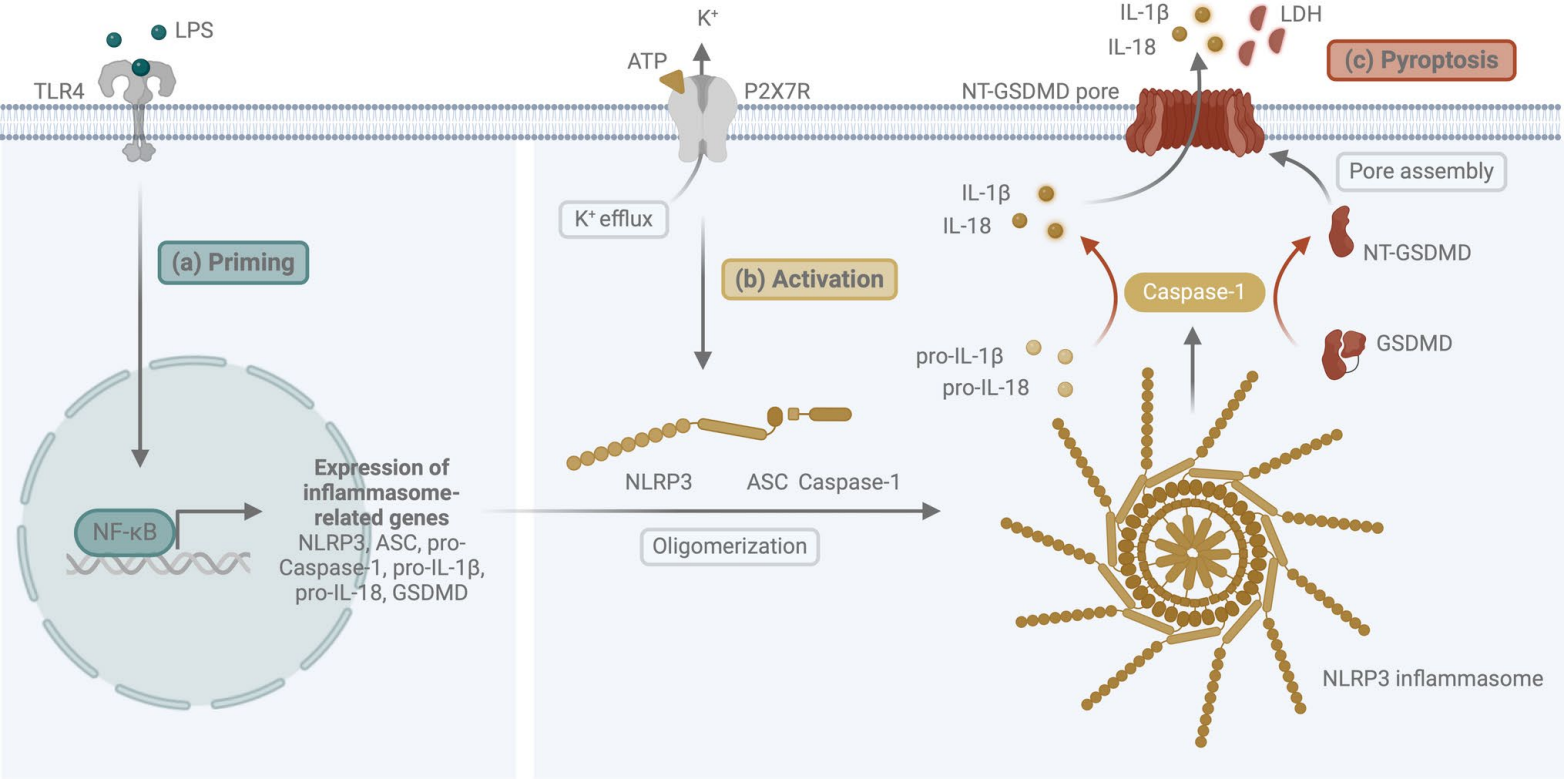
Canonical activation of the NLRP3 inflammasome. (**a**) First, priming is initiated by LPS via TLR4, resulting in the activation of the NF-κB pathway. Consequently, the expression of NLRP3, ASC, and the pro-forms of Casp-1, IL-1β, IL-18 and GSDMD is induced. (**b**) A second stimulus with ATP causes oligomerization of the NLRP3 inflammasome complex and the cleavage of Casp-1, the respective active interleukins and NT-GSDMD. (**c**) In turn, the NT-GSDMD form pores to release the matured interleukins and to provoke pyroptosis. *Abbreviations:* apoptosis-associated speck-like protein containing a caspase-recruitment domain (ASC); adenosine triphosphate (ATP); caspase-1 (Casp-1); gasdermin D (GSDMD); interleukin (IL); lactate dehydrogenase (LDH); lipopolysaccharide (LPS); nuclear factor 'κ-light-chain-enhancer' of activated B-cells (NF-κB); nucleotide-binding domain and leucine-rich repeat pyrin domain 3 (NLRP3); N-terminal (NT); P2X purinoreceptor 4/7 (P2XR4/7); toll-like receptor 4 (TLR4). Created with BioRender.com

The NLRP3 inflammasome is activated in a two-step process. This enables the regulation of important inflammatory mediators, such as Casp-1, interleukin (IL)−1β and IL-18. The initial signal, known as priming (Fig. 1a), is used for the transcriptional upregulation of NLRP3 inflammasome-associated genes and is triggered by cytokines or pathogen-associated molecular patterns (PAMPs), such as lipopolysaccharides (LPS). The transcription factor nuclear factor 'ĸ-light-chain-enhancer' of activated B-cells (NF-ĸB) plays a central role in this cascade as a key upstream regulator. During the second signal, known as activation (Fig. 1b), the oligomerization of the multiprotein complex NLRP3 is ensured. The activation is caused by damage-associated molecular patterns (DAMPs), such as adenosine triphosphate (ATP). Once the NLRP3 complex is formed, Casp-1 undergoes autoproteolytic cleavage [3, 4]. The mature Casp-1 fragment (p20) cleaves specifically the pro-forms of the pro-inflammatory cytokines IL-1β and IL-18 as well as the pyroptosis-associated protein gasdermin D (GSDMD). Pyroptosis (Fig. 1c) is the cell death initiated by an inflammasome. Subsequently, the N-terminal GSDMD (NT-GSDMD) fragment can bind to the plasma membrane and forms pores through oligomerization. This process releases not only mature IL-1β and IL-18, but also DAMPs, thereby initiating a potent local inflammatory response [6, 7]. Studies show that the NLRP3 inflammasome and inflammation-based diseases, such as Alzheimer disease, atherosclerosis, type 2 diabetes, and liver diseases, are closely linked [2].

In African folk medicine, the seeds of *Garcinia kola* E. Heckel (African bitternut) are known for diverse health-promoting properties. For example, it is used in anti-microbial, anti-viral, anti-diabetic and hepatoprotective therapies [8]. The presence of several bioactive compounds in these seeds, in particular garcinoic acid (GA), makes it an interesting source for the study of putative pharmacological activities. First, the antioxidant properties of GA were demonstrated in in vitro experiments [9, 10], followed by its potential anti-carcinogenic properties [11, 12]. Most recently, a study in LPS-stimulated murine RAW264.7 macrophages showed that GA also acts as an inhibitor of the pro-inflammatory proteins cyclooxygenase 2 (COX-2) and inducible nitric oxide synthase (iNOS) [13]. However, the mechanisms responsible for the anti-inflammatory effects have not yet been fully understood. As a tocotrienol (T3) derivative, GA is structurally a metabolite of δ-tocotrienol (*trans*−13′-carboxy-δ-tocotrienol, Fig. 2). Anti-inflammatory, anti-carcinogenic, cardio- and neuroprotective effects have been described for T3s in vitro, as well as anti-diabetic, immunomodulatory or gastroprotective effects in animal models [14, 15].

**Fig. 2:**

Structure of the vitamin E derivative GA (*trans*−13′-carboxy-δ-tocotrienol)

In relation to the NLRP3 inflammasome pathways, initial studies show protective effects of T3 [16, 17]. Therefore, we investigated the anti-inflammatory potential of the T3 derivative GA in J774A.1 macrophages activated by LPS only or in combination with ATP. Specifically, our research focused on the effects of GA on the canonical NLRP3 inflammasome signaling pathway.

## Results

### Effects of GA on the Priming of Murine Macrophages

#### GA Blocks the LPS-Induced Expression of Nlrp3 and Interleukins

**Fig. 3:**
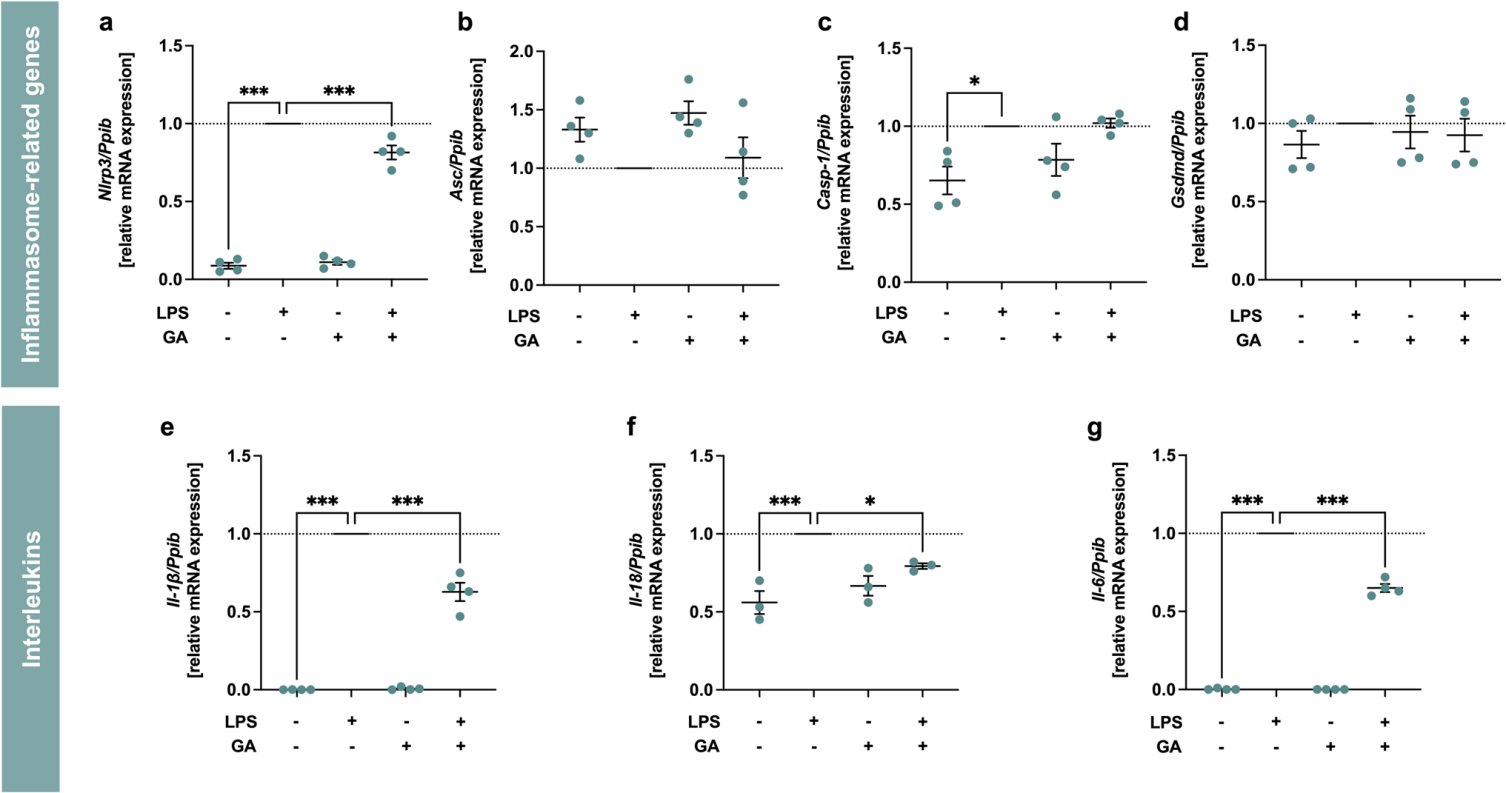
Effect of garcinoic acid (GA) on the expression of genes involved in the NLRP3 inflammasome pathway (**a**-**d**) and the cytokines *Il-1β, Il-18* and *Il-6* (**e**–**g**). Murine J774A.1 macrophages were treated with LPS (100 ng/ml) and/or GA (5 µM) for 4 h. The mRNA level was determined by RT-qPCR and normalized to the expression of the reference gene *Ppib*, which is constantly expressed under all conditions (Fig. 9 in the Supplementary Material). *Statistics:* *n* = 3 (**f**) or n = 4 (**a-e, g**); means ± SEM; one-way Anova with Dunnett’s post-hoc test. ^*^p < 0.05; ^***^p < 0.001 (vs. LPS). *Abbreviations:* apoptosis-associated speck-like protein containing a caspase-recruitment domain (Asc); caspase-1 (Casp-1); garcinoic acid (GA); gasdermin D (Gsdmd); interleukin (Il); lipopolysaccharide (LPS); nucleotide-binding domain and leucine-rich repeat pyrin domain 3 (Nlrp3); peptidylprolyl isomerase B (Ppib)

The first step in the NLRP3 signaling pathway is the priming, whereby inflammasome-relevant genes are expressed and regulated. Therefore, we investigated the effects of GA on LPS-induced inflammatory response in murine J774A.1 macrophages. We analyzed the effect of GA on the expression of NLRP3 inflammasome-related genes such as *Nlrp3, Asc, Casp-1* and *Gsdmd* (Fig. 3a-d). GA did not affect basal expression levels of these genes. Significant stimulation of mRNA expression by LPS was observed for *Nlrp3* (*p* < 0.001) and *Casp-1* (*p* < 0.05) compared to control. GA (5 µM) significantly decreased LPS-induced *Nlrp3* mRNA expression by 18% compared to LPS alone (Fig. 3a, p < 0.001). The mRNA expression of the interleukins *Il-1β, Il-18* and *Il-6* (Fig. 3e-g), which are pro-inflammatory mediators, were also measured. As expected, LPS significantly induced the expression of the genes of interest (*p* < 0.001), whereas GA alone had no effect on basal mRNA expression. GA (5 μM) blocked the expression of LPS-responsive genes *Il-1β, Il-18* and *Il-6* by 37% (*p* < 0.001), 21% (*p* < 0.05) and 35% (*p* < 0.001), respectively.

#### GA Affects NF-κB p65 Binding Activity

**Fig. 4:**
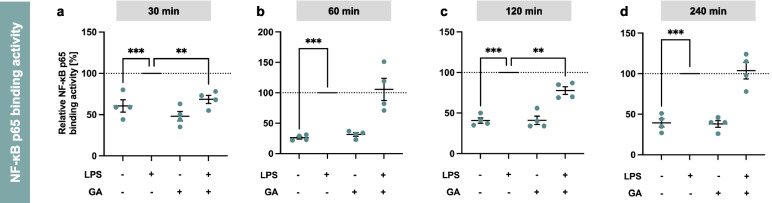
Modulation of NF-κB p65 binding activity in nuclear extracts by garcinoic acid (GA). Murine J774A.1 macrophages were treated with LPS (100 ng/ml) and/or GA (5 µM) for 30 min (**a**), 60 min (**b**), 120 min (**c**) and 240 min (**d**). The NF-κB p65 binding activity was determined by an enzyme-linked immunosorbent assay and normalized to protein level. * Statistics:* *n* = 4; means ± SEM; one-way Anova with Dunnett’s post-hoc test. ^**^p < 0.01; ^***^p < 0.001 (vs. LPS). * Abbreviations:* garcinoic acid (GA); lipopolysaccharide (LPS); nuclear factor 'κ-light-chain-enhancer' of activated B-cells (NF-κB)

Based on the initial results on mRNA gene expression for NLRP3 inflammasome-related genes and interleukins, we wanted to find out whether this regulation is controlled by the upstream transcription factor NF-κB. For this purpose, nuclear and cytosolic fractions of the cell samples were separated. Subsequently, the NF-κB p65 binding activity in the nuclear extract was determined by an enzyme-linked immunosorbent assay. The p65 protein is one of the most abundant and best-studied subunits and plays a crucial role in the activation of NF-κB. LPS-stimulation for 30 min, 60 min, 120 min and 240 min achieved the expected significant upregulation of NF-κB p65 binding activity at all time points compared to control (Fig. 4a-d, p < 0.001). Co-incubation with GA (5 µM) significantly reduced LPS-induced NF-κB p65 binding activity to 69 ± 5% (*p* < 0.01) at 30 min (Fig. 4a) and 78 ± 5% (*p* < 0.01) at 120 min (Fig. 4c).

### Effects of GA on the Activation of Murine Macrophages

#### GA does not Affect Nlrp3 Protein but Regulates the Cleavage of Casp-1

**Fig. 5:**
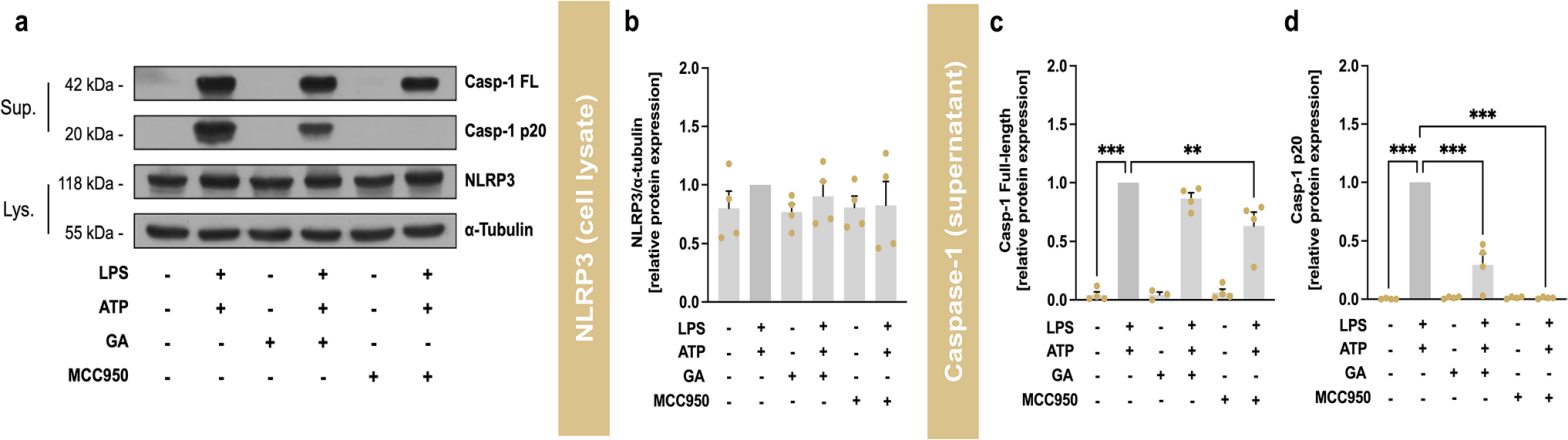
Effects of garcinoic acid (GA) on the priming and activation of murine J774A.1 macrophages. Representative western blots of cell lysates and supernatants from J774A.1 stimulated with LPS and ATP and treated with GA or MCC950 (**a**). These results are representative of four independent experiments. Relative expression of NLRP3 protein (**b**), Casp-1 full-length protein (**c**) and cleaved Casp-1 p20 fragment (**d**). J774A.1 macrophages were co-stimulated with LPS (100 ng/ml) for 4 h and another 2 h with ATP (3 mM). Next, the cells were incubated with GA (5 µM) or MCC950 (1 µM) as indicated in the figure legends. The signals of NLRP3 were normalized to the signal of the reference protein α-tubulin (Fig. 10 in the Supplementary Material). *Statistics:* *n* = 4; means ± SEM; one-way Anova with Dunnett’s post-hoc test. ^**^p < 0.01; ^***^p < 0.001 (vs. LPS + ATP). *Abbreviations:* adenosine triphosphate (ATP); caspase-1 (Casp-1); garcinoic acid (GA); lipopolysaccharide (LPS); cell lysate (Lys); inhibitor of the nucleotide-binding domain and leucine-rich repeat pyrin domain 3 inflammasome (MCC950); nucleotide-binding domain and leucine-rich repeat pyrin domain 3 (NLRP3); supernatant (Sup)

Co-incubation of LPS and ATP was used to activate the multiprotein complex NLRP3. MCC950 is a specific NLRP3 inhibitor that prevents the oligomerization of the multiprotein complex through direct interaction with the Walker B motif of NLRP3 and in turn the blocking of ATP hydrolysis [18] and was therefore used as reference compound with a concentration of 1 µM. To determine whether the observed mRNA expression changes are reproducible at the post-translational level, Western blot analysis of cell lysates and cell culture supernatants were performed. The expression of NLRP3 protein in cell lysates did not increase after stimulation with LPS and ATP compared to control (Fig. 5b). In addition, other stimulations or co-stimulations with GA or MCC950 had no influence on NLRP3 protein levels.

Next, we focused on Casp-1, which is considered as a key regulator in the entire NLRP3 signaling pathway. On the one hand, the presence of the active p20 fragment of Casp-1 proves the successful activation of the NLRP3 complex and consequently the autoproteolytic cleavage of the pro-form. On the other hand, the actived Casp-1 fragment p20 promotes the cleavage of interleukins IL-1β and IL-18 pro-forms into their mature forms (IL-1β p17 and IL-18 p18) and induces the pro-inflammatory response [4]. Upon activation, the Casp-1 fragment p20 is rapidly released into the cell culture supernatant [19], which is therefore used for Western blot analysis. To study the Casp-1 protein, macrophages were stimulated with LPS and ATP and/or co-incubated with GA (5 µM) or MCC950 (1 µM). Stimulation with LPS and ATP significantly increased (*p* < 0.001) the full-length Casp-1 protein (≈42 kDa, Fig. 5c) compared to control. While GA had no influence after co-incubation with LPS and ATP, MCC950 reduced the amount of Casp-1 full-length protein to 0.63 ± 0.12 (*p* < 0.01). More important, autoproteolytic cleavage of the Casp-1 fragment p20 (≈20 kDa, Fig. 5d) was induced by stimulation with LPS/ATP compared to control (*p* < 0.001). GA significantly reduced the secretion of the Casp-1 fragment p20 to 0.30 ± 0.10 (*p* < 0.001). No regulation of the full-length Casp-1 protein was observed in the cell lysates either after stimulation with LPS or in combination with LPS and ATP compared to the control (Fig. 17 in the Supplementary Material). As expected, the cleavage of Casp-1 p20 was nearly completely prevented by MCC950 (0.01 ± 0.004, p < 0.001), as the NLRP3 complex failed to successfully oligomerize.

#### GA Reduces Cleavage Of Interleukins IL-1β and IL-18

**Fig. 6:**
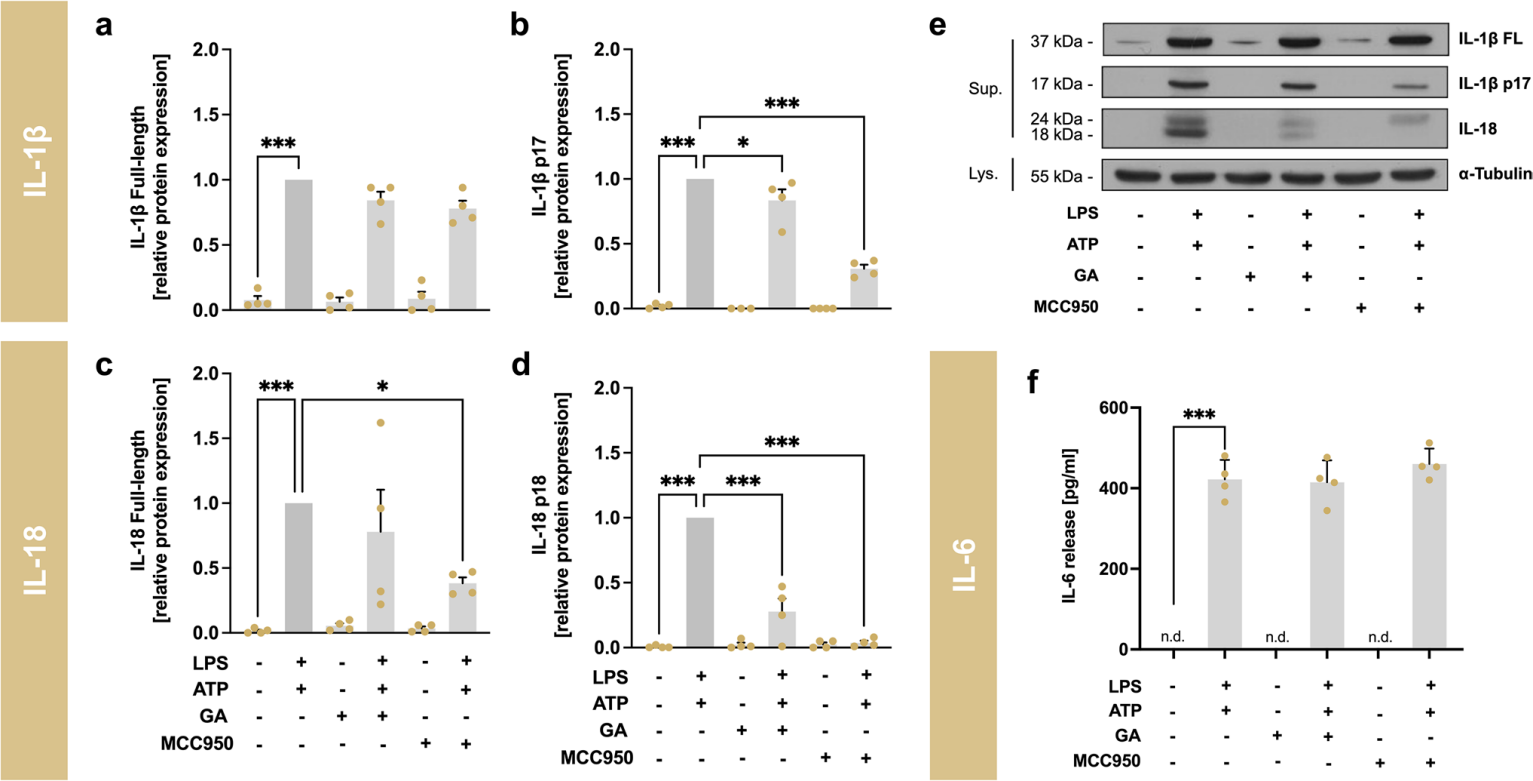
Effects of garcinoic acid (GA) on releasing and cleavage of interleukins. Relative expression of the cytokines IL-1β and IL-18 (**a** and **c**) and their cleaved fragments p17 (**b**) as well as p18 (**d**) were measured in the cell culture supernatant using Western blotting. Representative western blots of supernatants from J774A.1 stimulated with LPS and ATP and treated with GA or MCC950 (**e**). These results are representative of four independent experiments. Release of IL-6 (**f**) in cell culture supernatant was determined by an enzyme-linked immunosorbent assay. J774A.1 macrophages were co-stimulated with LPS (100 ng/ml) for 4 h followed by 2 h with ATP (3 mM). Further the cells were incubated with GA (5 µM) or MCC950 (1 µM) as indicated in the figure. *Statistics:* *n* = 4; means ± SEM; one-way Anova with Dunnett’s post-hoc test. ^*^p < 0.05; ^***^p < 0.001 (vs. LPS + ATP). *Abbreviations:* adenosine triphosphate (ATP); garcinoic acid (GA); interleukin (IL); lipopolysaccharide (LPS); cell lysate (Lys); inhibitor of the nucleotide-binding domain and leucine-rich repeat pyrin domain 3 inflammasome (MCC950); not detectable (n.d.), supernatant (Sup)

Cleavage of Casp-1 further cleaves the pro-inflammatory interleukins IL-1β and IL-18 into their mature forms (IL-1β p17 and IL-18 p18). To investigate the influence of GA on this cleavage, J774A.1 macrophages were stimulated with LPS and ATP and co-incubated with GA (5 µM), as indicated in the legend of Fig. 6. MCC950 (1 µM) was used as reference compound. Respective cell culture supernatants were used to determine IL release and formation of the mature forms (Fig. 6a-d). In cells stimulated with LPS and ATP both the full-length protein of IL-1β (≈37 kDa, Fig. 6a, *p* < 0.001) as well as the p17 fragment (≈17 kDa, Fig. 6b, *p* < 0.001) were significantly upregulated. GA blocked the cleavage of the IL-1β p17 fragment (0.84 ± 0.09, *p* < 0.05) similar to MCC950 (0.31 ± 0.03, *p* < 0.001), whereas no significant changes of the full-length IL-1β protein were observed. A similar regulation of interleukin IL-18 was observed. Stimulation with LPS and ATP increased expression and cleavage of both the IL-18 full-length protein (≈24 kDa, Fig. 6c, p < 0.001) and the p18 fragment (≈18 kDa, Fig. 6d, *p* < 0.001). MCC950 significantly reduced IL-18 full-length protein to 0.38 ± 0.05 (*p* < 0.05) and the p18 fragment to 0.04 ± 0.02 (*p* < 0.001) in stimulated cells. In contrast, GA had no significant effect on the IL-18 full-length protein. However, a 72% (0.28 ± 0.10, *p* < 0.001) lower protein amount was detected for the cleavage product of IL-18, p18 fragment, compared to stimulated cells. Incubation with LPS and ATP resulted in a significant increase in the release of IL-6 (*p* < 0.001), which was not augmented by either GA or MCC950 (Fig. 6f).

### Effects of GA on Pyroptosis

#### GA Attenuates GSDMD Cleavage

**Fig. 7:**
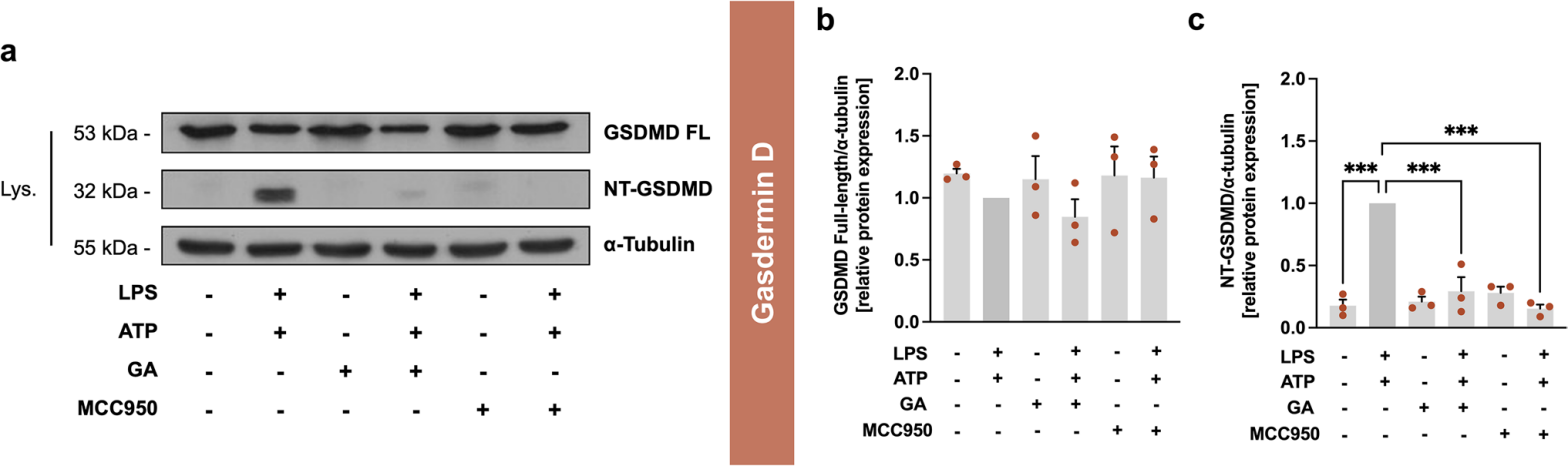
Effects of garcinoic acid (GA) on the pyroptosis-associated protein GSDMD. Representative western blots of cell lysates from J774A.1 stimulated with LPS and ATP and treated with GA or MCC950 (**a**). These results are representative of three independent experiments. Relative expression of GSDMD protein in full-length (**b**) and cleaved NT-GSDMD (**c**). J774A.1 macrophages were co-stimulated with LPS (100 ng/ml) for 4 h and ATP (3 mM) for 2 h. Further the cells were incubated with GA (5 µM) or MCC950 (1 µM) as indicated in the figure. The signals of GSDMD full-length and NT-GSDMD were normalized to the signal of the reference protein α-tubulin (Fig. 10 in the Supplementary Material). *Statistics:* *n* = 3; means ± SEM; one-way Anova with Dunnett’s post hoc test. ^***^p < 0.001 (vs. LPS + ATP). *Abbreviations:* adenosine triphosphate (ATP); garcinoic acid (GA); gasdermin D (GSDMD); lipopolysaccharide (LPS); cell lysate (Lys); inhibitor of the nucleotide-binding domain and leucine-rich repeat pyrin domain 3 inflammasome (MCC950); N-terminal (NT)

GSDMD, and specifically its cleavage product NT-GSDMD, plays a crucial role in pyroptotic cell death by forming a pore in the plasma membrane after cleavage. This pore allows for the release of mature fragments of IL-1β and IL-18, which can increase the local inflammatory reaction [7]. For the full-length GSDMD protein (≈53 kDa, Fig. 7b), no significant change in protein expression was observed under the indicated incubation conditions. As expected, treatment with LPS and ATP increased the cleavage of NT-GSDMD (≈32 kDa, Fig. 7c), compared to control (*p* < 0.001), which was reduced by GA to 0.29 ± 0.11 (*p* < 0.001). The control substance MCC950 also reduced the effect size of LPS/ATP to 0.15 ± 0.03 (*p* < 0.001).

#### GA Mitigates Hallmarks of Pyroptosis

**Fig. 8:**
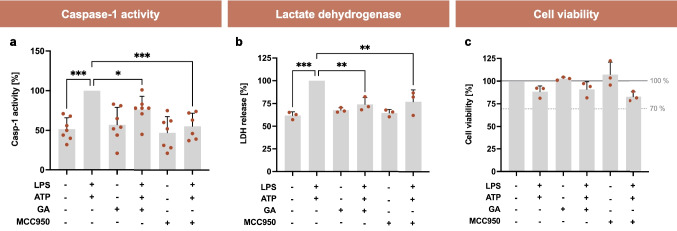
Effects of garcinoic acid (GA) on hallmarks of pyroptosis. Casp-1 activity (**a**), release of LDH (**b**), and cell viability (**c**) were measured using different photometric assays. J774A.1 macrophages were co-stimulated with LPS (100 ng/ml) for 4 h followed by 2 h with ATP (3 mM). Next, the cells were incubated with GA (5 µM) or MCC950 (1 µM) as indicated in the figure. The measurements of LDH release (**b**) were normalized to the cell viability rate of the respective biological replicate and were always above the viability limit of 70% [22]. *Statistics:* *n* = 3 (**b** and **c**) or 7 (**a**); means ± SEM; one-way Anova with Dunnett’s post-hoc test. ^*^p < 0.05; ^**^p < 0.01; ^***^p < 0.001 (vs. LPS + ATP (**a** and **b**) or control (**c**)). *Abbreviations:* adenosine triphosphate (ATP); caspase-1 (Casp-1); garcinoic acid (GA); lactate dehydrogenase (LDH); lipopolysaccharide (LPS); inhibitor of the nucleotide-binding domain and leucine-rich repeat pyrin domain 3 inflammasome (MCC950)

Since there is no direct measurement of the inflammasome-specific cell death (pyroptosis), a combination of the following markers are used for evaluation: GSDMD cleavage (Fig. 7), Casp-1 activity, lactate dehydrogenase (LDH) release, and cell viability [20]. As expected, stimulation with LPS and ATP induced Casp-1 activity by approximately 100% (*p* < 0.001, Fig. 8a). Both, GA and MCC950 reduced LPS/ATP-induced Casp-1 activity to 76 ± 6% (*p* < 0.05) and 55 ± 7% (*p* < 0.001), respectively, compared to stimulated control. LDH release correlates with cell damage and cytotoxicity, making it a suitable marker of cell death. Treatment with LPS and ATP increased LDH release by approximately 60% (*p* < 0.001, Fig. 8b). GA (26%, *p* < 0.01) and MCC950 (23%, *p* < 0.01) were equally effective in reducing LPS/ATP-induced release of LDH compared to the stimulation with LPS and ATP only. To demonstrate that observed effects are independent of possible cytotoxic effects of our compound of interest, the impact of GA on cell viability was determined. The requirement for the classification of a substance as non-cytotoxic is a cell viability of at least 70% [21]. Neither the stimulation with LPS and ATP nor the treatment with GA and MCC950 for 6 h in total significantly affected cell viability compared to the control (Fig. 8c). The release of LDH is often accompanied by cell damage and lysis. The unchanged cell viability suggests that transient membrane damage causes LDH release without direct cell death. Sensitive LDH measurement also accounts for premature release that includes reparable damage.

## Discussion

An innovative strategy for treating inflammation-related diseases is the identification of novel therapeutic agents, such as phytochemicals. *Garcinia kola* is used in African folk medicine for the treatment of inflammation [23]. Of particular interest are the seeds from *Garcinia kola*, which contain the phytochemical GA as a major component that exhibits anti-inflammatory effects in vitro [13, 24–27]. In addition, in vitro studies with murine macrophages have demonstrated the anti-inflammatory potential of T3 [22, 28], which are structurally related to GA. However, the mechanistic processes underlying the observed effects are unknown.

Therefore, the aim of this study was to elucidate the effects of GA on a key regulator of inflammation, the NLRP3 inflammasome. To achieve this, we investigated the three levels of regulation of the NLPR3 in murine J774A.1 macrophages: (i) priming, (ii) activation of the NLRP3 complex, and (iii) pyroptosis using the canonical activation with LPS alone or in combination with ATP.

### Effects of Garcinoic Acid on Macrophage Priming

We used LPS to stimulate the priming of the inflammasome NLRP3. Stimulation with LPS upregulated the interleukins *Il-1β, Il-18*, and *Il-6*, whereas co-stimulation with GA significantly blocked the upregulation of expression. These findings confirm the anti-inflammatory effect of GA previously observed by Wallert and colleagues at the gene regulatory level [13]. They reported a significant downregulation of pro-inflammatory genes, such as *tumor necrosis factor (Tnf)-α, Il-1β, Il-6, Cox2*, and *iNos* in murine RAW264.7 macrophages after stimulation with LPS by GA (1 to 5 µM). The RAW264.7 macrophage cell line, which is commonly used to study inflammatory processes, does not express a functional ASC complex to activate the NLRP3 inflammasome [29, 30]. Therefore, the RAW264.7 cell line can only be used to study the priming level, i.e. the state before oligomerization of the NLRP3 complex. Since we wanted to generate a comprehensive insight from priming to pyroptosis, we only used the J774A.1 cell line for our study. A previous study was conducted in murine RAW264.7 macrophages to investigate the effect of γ-T3 (1 µM) and δ-T3 (1 to 5 µM) on LPS stimulation at the priming level [31]. The study revealed a suppression of mRNA expression of *Nlrp3, Tnf-α, Il-1β,* and *Il-18*. In another in vitro study, mouse bone marrow-derived macrophages (BMDMs) were pre-treated with 1 µM γ-T3 for 24 h, followed by NLRP3 inflammasome stimulation with LPS and nigericin [16]. Nigericin is an alternative ATP stimulator of NLRP3 activation; both ATP and nigericin induce potassium efflux, thereby triggering the activation and assembly of the NLRP3 complex in the same way [32, 33]. As demonstrated in the study by Kim et al*.*, the mRNA expression of Il-1β, Il-18 and Nlrp3 was significantly reduced by γ-T3 [16]. Gene expression does not always directly correlate with changes at the protein level or functional outcomes. Therefore, we conducted further studies at the protein level to evaluate regulatory effects in Casp-1, interleukin, and GSDMD protein expression (see chapters "Effects of Garcinoic Acid on Macrophage Activation" and "Effects of Garcinoic Acid on Pyroptosis" in the discussion for the respective results).

The effects of GA on the analyzed cytokines are supported by the investigation of NF-κB p65 binding activity, which is significantly increased after stimulation with LPS and augmented by a co-treatment with GA (Fig. 4a and c). This is relevant as the cytokines we investigated are regulated by the upstream transcription factor NF-κB [3]. This is in line with previous experiments, in which Olajide and colleagues [24] stimulated human peripheral blood mononuclear cells (PBMCs) with the spike protein S1 the severe acute respiratory syndrome coronavirus type 2 (SARS-CoV-2), which resulted in a strong induction of NF-κB binding activity. Co-incubation with GA (1.25 to 5 µM) significantly reduced the NF-κB binding activity. Our results suggest, that GA, at least partly, blocks NF-κB-dependent expression of NLRP3 inflammasome-related genes. Furthermore, there is evidence that the structurally related molecule γ-T3 is also a potent inhibitor of NF-κB activity [16, 34, 35].

### Effects of Garcinoic Acid on Macrophage Activation

In our experiments, activation and oligomerization of the NLRP3 complex were initiated by co-stimulation with LPS and ATP. The stimulation resulted in a significant activation of the NLRP3 inflammasome at the protein level (Casp-1, IL-1β, IL-18 and IL-6; Fig. 5c-d and 6). The expression of cleaved Casp-1 p20 after stimulation was significantly decreased by GA (Fig. 5d). In line with this, Buckner and colleagues [31] showed that δ-T3 obtained from annatto (1 to 5 µM) reduced the activation of the NLRP3 inflammasome in murine J774A.1 macrophages as shown by the decreased IL-1β reporter activity, IL-1β secretion, and Casp-1 cleavage in response to LPS and nigericin. Another study investigated the effect of 1 µM γ-T3 on the NLRP3 inflammasome in J774A macrophages and in BMDMs stimulated with LPS and nigericin [16]. The study showed a decrease in Casp-1 cleavage and IL-1β secretion in J774 macrophages. These findings were confirmed in BMDMs, where treatment with γ-T3 suppressed the secretion of IL-1β and IL-18. Cleavage of Casp-1 and NLRP3 protein expression were also downregulated in LPS-primed and nigericin-stimulated cells [16]. In human differentiated THP-1 macrophages, a nitroalkene vitamin E analog (NATx0) regulated the activation of the NLRP3 inflammasome [36]. After co-incubation with LPS and ATP, NATx0 reduced IL-1β secretion.

Furthermore, we found that GA inhibits pro-inflammatory cytokines, such as IL-1β and IL-18, in stimulated cells (Fig. 6b and d). Next to NLRP3-dependent formation of IL-1β, alternative secretion pathways for IL-1β are known [37]. Thus, regulation of IL-1β is also detectable in RAW264.7 macrophages, despite insufficient NLRP3 complexation [38]. Furthermore, IL-6 is a crucial marker for assessing systemic inflammation as a downstream target of IL-1β and a target gene of NF-κB [3]. We found that LPS/ATP-stimulated expression of IL-6 mRNA was blocked by GA, whereas the protein expression remained unchanged (Fig. 3g and 6f). Serum IL-6 levels are elevated in humans [39] and mice [40], with NLRP3 inflammasome-mediated diseases, such as cryopyrin-associated periodic syndromes (CAPS), a disease caused by mutations in the *Nlrp3* gene [41]. However, a study in mice has shown that IL-6 levels are not always associated with NLRP3 inflammasome activation and therefore, IL-6 is not a specific therapeutic target for NLRP3 inflammasome-related diseases [42]. This finding shows the possible gap in the regulation of NLRP3-dependent and -independent inflammation markers and could clarify the lack of regulation of IL-6 at the protein level following stimulation with LPS and ATP and co-incubation with GA in our study (Fig. 6f). However, our findings suggested that GA inhibits the NLRP3 inflammasome activation.

### Effects of Garcinoic Acid on Pyroptosis

Pyroptosis is a form of Casp-1-dependent cell death that results in a loss of membrane integrity, leading to fluid influx, cell swelling, and lysis, and is triggered by the activation of inflammasomes [6, 43]. Casp-1 activity serves as a crucial marker for the assessment of pyroptosis [44, 45]. In our study, GA significantly reduced Casp-1 activity (Fig. 8a), cleavage of NT-GSDMD (Fig. 7c), and release of LDH in LPS/ATP-stimulated cells (Fig. 8b). Co-incubation with LPS and ATP in combination with NATx0 decreased markers of pyroptosis such as ASC formation and release of LDH in human THP-1 cells [36]. Our results indicate that GA significantly reduced pyroptosis induced by LPS and ATP. Taken together, our findings suggest that GA mediates anti-inflammatory mechanisms also through the NLRP3 inflammasome pathway, with clear evidence indicating that GA can significantly modulate key components of the NLRP3 inflammasome. Our study provides valuable insights into the anti-inflammatory properties of GA, particularly its role in modulating the NLRP3 inflammasome pathway.

However, several limitations warrant consideration. The experiments were conducted in a specific murine cell model, and the findings may not fully capture the complexity of physiological conditions in vivo. In addition, the focus on NLRP3 inflammasome activation via LPS and ATP stimulation leaves open the question of whether GA influences other activation pathways such as non-canonical or disease-specific activations (e.g. cholesterol crystals in atherosclerosis or lipid-induced activation in steatotic liver diseases) or other inflammasomes such as the absent in melanoma (AIM2) or NLR family CARD domain containing 4 (NLRC4) inflammasome. Future studies exploring diverse models and broader inflammasome interactions could further elucidate the scope and mechanisms of anti-inflammatory effects of GA. Despite these limitations, our findings represent a significant step forward in understanding the potential of GA as a natural modulator of inflammation.

In an earlier study, Kluge and colleagues [8] made initial assumptions about the efficiency of GA in inflammatory processes. They based this on the similar chroman ring structure of δ-TOH, which is considered to be the most effective TOH for inflammation. So far, there are no comparable in vitro studies on the effects of TOH on the regulation of the NLRP3 inflammasome. In addition, δ-T3 is more effective than other T3s in modulating pro-inflammatory parameters, such as COX activity. The unsaturated chain is a structural feature of GA that it shares with T3s. GA also acts as a starting point for the synthetic synthesis of the so-called long-chain vitamin E metabolites (LCMs) 13'-hydroxychromanol and 13'-carboxychromanol (13'-OH and 13'-COOH). The detection of these metabolites in human blood serum indicates their physiological relevance [46]. The protective effects of LCMs on lipid metabolism, apoptosis, proliferation and inflammatory processes have already been demonstrated [47]. Since GA serves as a lead structure of the LCMs, the investigation of the influence of the other LCMs on the NLRP3 inflammasome is of great interest for further studies.

## Material and Methods

### Chemicals

Unless otherwise indicated, the chemicals were obtained from Carl Roth (Karlsruhe, Germany), Merck (Darmstadt, Germany; includes Merck Millipore and Sigma-Aldrich) or Thermo Fisher Scientific (Darmstadt, Germany; includes Fermentas, Invitrogen and Fisher Scientific). The chemicals required for incubation are dissolved as follows: ATP and MCC950 in nuclease-free water, GA in dimethyl sulfoxide (DMSO) and LPS in phosphate buffered saline (PBS).

### Isolation of GA

The *Garcinia kola* E. Heckel seeds were used to isolate GA (*trans*−13′-carboxy-δ-tocotrienol) according to the protocol described by [12, 13].

### Cell Culture

Murine J774A.1 macrophages (400,220, CLS, Eppelheim, Germany) were cultivated in high-glucose Dulbecco's Modified Eagle's Medium (DMEM) cell culture medium supplemented with 10 mg/ml fetal bovine serum (FBS) and 0.1 mg/ml penicillin–streptomycin-glutamine (PSG) solution. Cells were cultured at 37 °C in a humidified 5% CO_2_/95% air atmosphere. For experiments cells were seeded in different densities, cultured for 24 h, grown to a confluence of approximately 80% and then incubated with FBS-free DMEM and the compounds as indicated in the figure legends. The cells were harvested, and the supernatant collected for further processing as described below.

### Isolation of Nuclear Extracts

To identify the binding activity of NF-κB p65, the the NE-PER™ Nuclear Cytoplasmic Extraction Reagent (78,835, Thermo Fisher Scientific) was used for isolation of the nuclear extract. We made slight modifications to the cell harvesting process: we removed the entire supernatant, washed the cells twice with warm PBS (37 °C), and then mechanically detached the cells with 500 μl PBS. We rinsed the cells with another 500 μl PBS and transferred the entire suspension to a reaction vessel. This is followed by centrifugation at 500 × g for 3 min at room temperature. The further isolation process is carried out according to the manufacturer's instructions.

### NF-κB p65 Transcription Factor Assay

The nuclear fraction was used for the NF-κB p65 Transcription Factor Assay (133,112, Abcam, Cambridge, UK) according to the manufacturer’s instructions. Protein content of the nuclear extracts was determined with the Pierce BCA Protein Assay Kit (23,227, Thermo Fisher Scientific). Procedure was in accordance with the manufacturer's protocol.

### RNA Isolation, cDNA Synthesis and Quantitative Real-Time PCR (RT-qPCR)

Total RNA was isolated with the RNeasy Mini Kit (74,106, Qiagen, Hilden, Germany) as described in [48]. The cDNA synthesis was performed using Revert Aid First Strand cDNA Synthesis Kit (K1622, Thermo Fisher Scientific) and 500 ng/μl oligo-dT primers as described [49]. The primer pairs were purchased from Thermo Fisher Scientific, and their sequences are provided in Tab. 1 in the Supplementary Material. All primers were tested for efficiency. RT-qPCR analysis was performed on a LightCycler 480 II instrument (Hoffmann-La Roche, Basel, Switzerland) according to the methodology described in [50]. LightCycler software version 1.5.0.39 (Roche Diagnostics, Mannheim, Germany) was used to examine the PCR results.

### Immunoblotting

#### Sample Preparation

##### Cell Lysate

For the detection of proteins in the cell fraction, cells were harvested using a nondenaturing buffer (50 mM Tris–HCl, 0.5% Nonidet P40, 250 mM NaCl, 15 mM EDTA, 50 mM NaF, 0.5 mM Na_3_VO_4_). Moreover, it was supplemented with 1% protease inhibitor and mixed in a 3:1 ratio with SDS sample buffer consisting of 6.26% 1 M Tris–HCl, 2.3% (w/v) SDS, 10% glycerol, 5% 2-mercaptoethanol, and 0.1% bromophenol blue. Before applying the samples to the acrylamide gel (see details in Tab. 2 in the Supplementary Material), samples were sonificated for 5 s, heated at 95 °C for 5 min and centrifuged at 10,000 × g for 5 min at 4 °C.

##### Cell Culture Supernatant

Methanol-chloroform protein precipitation was performed according to the protocol of Wessel and Flügge [51] with slight modifications as described in [52].

#### Western Blotting

The proteins underwent separation via SDS-PAGE using an acrylamide gel (see details in Tab. 2 in the Supplementary Material) and were transferred to a PVDF membrane using a transfer buffer containing 0.25 M Tris, 1.92 M glycine, 0.1% (w/v) SDS and 20% methanol (pH 8.3). For protein detection, primary antibodies against Casp-1 (AG-20B-0042, Biomol, Hamburg, Germany), GSDMD (ab209845, Abcam), IL-1β (ab234437, Abcam), IL-18 (ab207323, Abcam), NLRP3 (ab270449, Abcam) and α-tubulin (T5168, Merck) as well as secondary antibodies (horseradish peroxidase-labelled swine anti-rabbit and rabbit anti-mouse) were used (DAKO, Hamburg, Germany; 1:5000). To enhance the chemiluminescence signal of Casp-1, GSDMD, IL-1β, IL-18 and NLRP3 antibodies, the Pierce Western Blot Signal Enhancer (21,050, Thermo Fisher Scientific) was utilized. Meanwhile, the α-tubulin antibody was incubated in a hybridization buffer containing 0.5% milk powder in PBS (0.137 M NaCl, 2.7 mM KCl, 6.5 mM Na_2_HPO_4_ × 2 H_2_O, 1.5 mM KH_2_PO_4_, pH 7.4) or TBS (20 mM Tris, 0.137 M NaCl, pH 7.4). Details for the primary antibodies (Casp-1, GSDMD, IL-1β, IL-18, NLRP3 and α-tubulin), along with their respective conditions, are summarized in Tab. 2 in the Supplementary Material. For detection, Pierce ECL Western Blotting Substrate (32,106, Thermo Fisher Scientific) and CL-XPosure Films (34,091, Thermo Fisher Scientific) were applied. The exposure times for Casp-1, GSDMD, IL-1β, IL-18, NLRP3 and α-tubulin are summarized in Tab. 2 in the Supplementary Material. The blots were analyzed by densitometry using the GelAnalyzer software, version 19.1.

### ELISA

To measure the amount of mouse IL-6 released in the supernatant, we used the commercially available IL-6 Mouse Uncoated ELISA Kit (88–7064-22, Thermo Fisher Scientific) according to the manufacturer's instructions. The IL-6 ELISA requires the ELISA Buffer Kit (CNB0011, Thermo Fisher Scientific) as an essential component.

### Measurement of Casp-1 Activity

The Casp-1 activity was quantified using the Caspase-Glo® 1 Inflammasome Assay (G9951, Promega, Madison, USA). Methodological modifications were made to enable measurement. A detailed description of these modifications can be found in [24].

### Measurement of LDH Release

The CytoTox 96 Non-Radioactive Cytotoxicity Assay (G1780, Promega) was used to investigate the release of LDH post-cell lysis. The cell culture supernatant was diluted 1:2 with water before measurement according to the manufacturer's instructions. The results were subsequently expressed as a percentage of LDH release.

### Cell Viability Measurement

After the incubation was completed, the cells were washed twice with FBS-free DMEM. The cells were then treated with 3-(4,5-dimethylthiazol-2-yl)−2,5-diphenyltetrazolium bromide (MTT, 2 mg/ml in PBS) at 50 μl per well in 500 μl FBS-free DMEM for 2 h at 37 °C. After removal of the cell culture medium, the resulting formazan was dissolved in 750 µl of isopropanol. The optical density was measured at 570 nm via the FLUOstar Omega microplate reader (BMG Labtech, Ortenberg, Germany), and viability units were normalized to the untreated control (100%).

### Statistical Analysis

Data is presented as mean ± SEM. All experiments were performed in three to seven independent biological replicates (for details see figure legends). To test for statistical significance, a one-way Anova with Dunnett’s post-hoc test was used. To identify outliers, a test for Gaussian normal distribution (Shapiro–Wilk test) and then the outlier test according to Grubbs (α = 0.05%) were performed. Statistics was implemented by using Prism 10 software version 10.1.0 (GraphPad Software, San Diego, USA).

## Supplementary Information

Below is the link to the electronic supplementary material.

Supplementary file1 (DOCX 625 KB)

## Data Availability

No datasets were generated or analysed during the current study.
