# Supplementary material for: The Vitamin E Derivative Garcinoic Acid Suppresses NLRP3 Inflammasome Activation and Pyroptosis in Murine Macrophages
Source: Inflammation. 2025 Feb 21;48(5):3340–52. doi: 10.1007/s10753-025-02269-6 (PMC12596286; doi:10.1007/s10753-025-02269-6)
Supplement: Supplementary file 1 — Supplementary file1 (DOCX 625 KB) [file 10753_2025_2269_MOESM1_ESM.docx]

**Supplementary Material**

The vitamin E derivative garcinoic acid suppresses NLRP3 inflammasome activation and pyroptosis in murine macrophages

Lisa Börmel, Anja R. Geisler, Yvonne Hupfer, Sijia Liao, Tina Schubert, Stefan Kluge, Stefan Lorkowski, Maria Wallert*

^*^Corresponding author

***Address for correspondence***

Dr. Maria Wallert

Institute of Nutritional Sciences

Friedrich Schiller University Jena

Dornburger Straße 25

07743 Jena

Germany

E-mail: maria.wallert@uni-jena.de

**Table 1:** Sequences of the PCR primers used in the study. The forward and reverse primers are located in different exons.

| **mRNA** | **mRNA name** | **Origin** | **GenBank accession no.** | **Forward primer** | **Reverse primer** |
| --- | --- | --- | --- | --- | --- |
| *Asc* | *PYD and CARD domain containing* | *Mus musculus* | NM_023258.4 | AACTGCGAGAAGGCTATGGG | TGGTCCACAAAGTGTCCTGTT |
| *Casp-1* | *Caspase-1* | *Mus musculus* | NM_009807.2 | ATTGCTTTCTGCTCTTCAACACC | CTCCAAGTCACAAGACCAGGC |
| *Gsdmd* | *Gasdermin D* | *Mus musculus* | NM_026960.4 | AAGGTTCTGGAAACCCCGTT | TGCCCTGAATGTTCCCATCG |
| *Il-1β* | *Interleukin-1β* | *Mus musculus* | NM_008361.4 | TGAAGTTGACGGACCCCAAA | CAGCCACAATGAGTGATACTGCC |
| *Il-6* | *Interleukin-6* | *Mus musculus* | NM_031168.2 | TCAATTCCAGAAACCGCTATGAA | GGAAGGCCGTGGTTGTCAC |
| *Il-18* | *Interleukin-18* | *Mus musculus* | NM_001357221.1 | TCTGCAACCTCCAGCATCAG | TCCTTGAAGTTGACGCAAGAGT |
| *Nlrp3* | *NLR family, pyrin domain-containing 3* | *Mus musculus* | NM_145827.4 | ATTGCTGTGTGTGGGACTGA | ACCAATGCGAGATCCTGACA |
| *Ppib* | *Peptidylprolyl isomerase B* | *Mus musculus* | NM_011149.2 | AAACAGCAAGTTCCATCGTGTCAT | GAAGCGCTCACCATAGATGCTCT |

**Table 2:** Details for primary antibodies used for immunoblotting.

| **Antibody** | **Distributor** | **Item number** | **Acrylamide gel** | **PVDF membrane** | **Concentration 1^st^ AB** | **Exposure time** |
| --- | --- | --- | --- | --- | --- | --- |
| **Cell fraction** | | | | | | |
| Anti-rabbit-GSDMD | Abcam | ab209845 | 10% | 0.45 μm^1^ | 1:1000 | 20 min |
| Anti-rabbit-NLRP3 | Abcam | ab270449 | 8% | 0.45 μm^1^ | 1:1000 | 15 min |
| Anti-mouse-α-Tubulin  (Reference protein) | Merck | T5168 | Depends on detected protein | 0.45 μm^1^ | 1:5000 | 1 min |
| **Supernatant** | | | | | | |
| Anti-mouse-Casp-1 | Biomol | AG-20B-0042 | 12% | 0.2 μm^2^ | 1:2000 | 10 min |
| Anti-rabbit-IL-1β | Abcam | ab234437 | 12% | 0.2 μm^2^ | 1:2000 | 25 min |
| Anti-rabbit-IL-18 | Abcam | ab207323 | 15% | 0.2 μm^2^ | 1:1000 | 10 min |

^1^ VWR International (Radnor, USA)

^2^ Merck (Darmstadt, Germany)


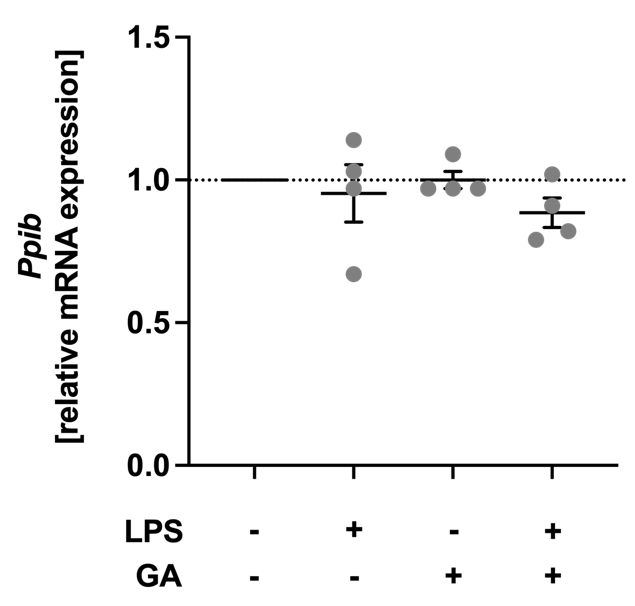


**Supplementary Figure 9**: Expression of the reference gene *Ppib*. This gene was used to normalize the expression levels of target genes shown in Figure 3. ***Statistics:*** n=4; means ± SEM; one-way Anova with Dunnett’s post-hoc test (vs. control).


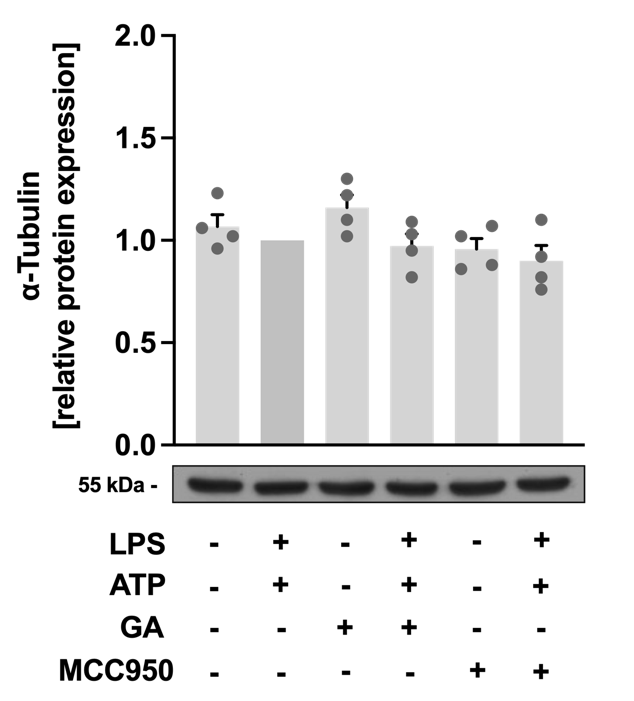


**Supplementary Figure 10**: Expression of the reference protein α-tubulin. This protein was used to normalize the expression levels of target proteins shown in Figures 5a and 7. ***Statistics:*** n=4; means ± SEM; one-way Anova with Dunnett’s post-hoc test (vs. control).


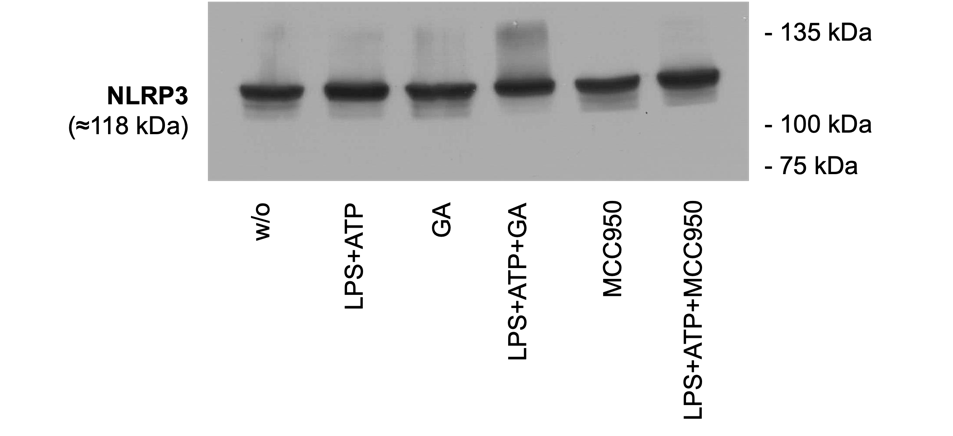


**Supplementary Figure 11**: Unprocessed Western Blot image of NLRP3 (cell lysate). The test conditions can be found in Table 2 of the supplement. Protein marker used: BlueEye Prestained Protein Marker (Jena BioScience).


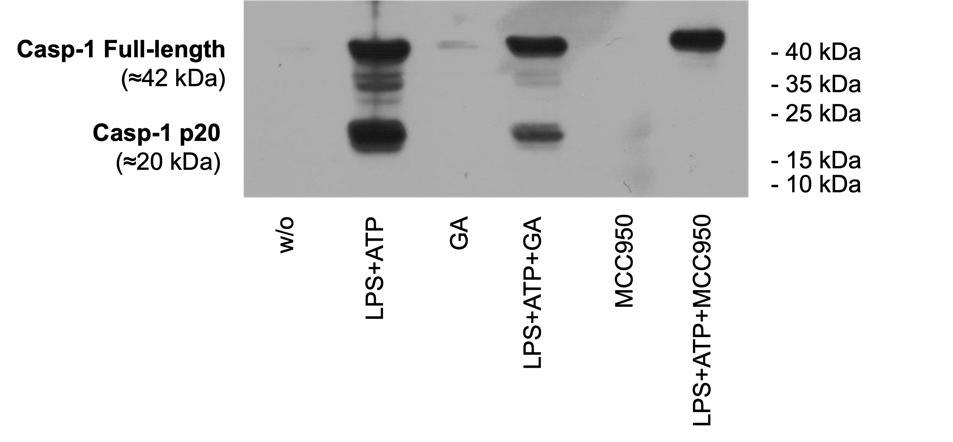


**Supplementary Figure 12**: Unprocessed Western Blot image of Casp-1 (supernatant). The test conditions can be found in Table 2 of the supplement. Protein marker used: PageRuler Prestained Protein Ladder (Thermo Scientific).


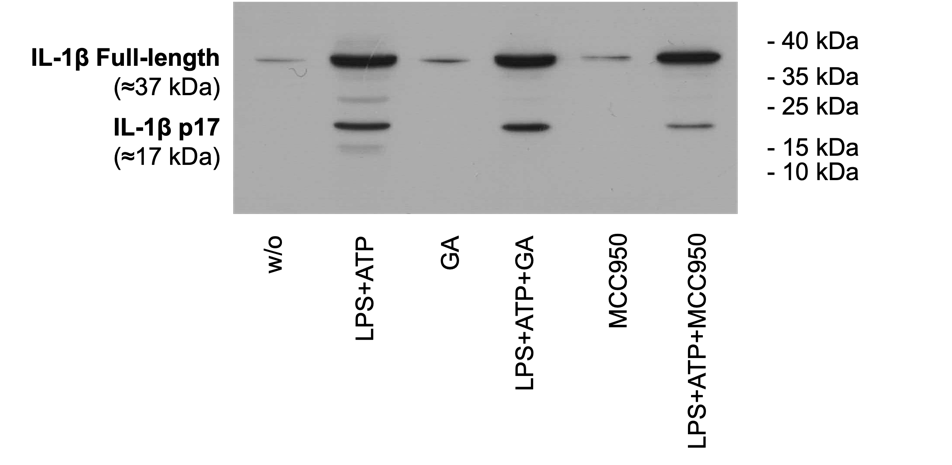


**Supplementary Figure 13**: Unprocessed Western Blot image of IL-1β (supernatant). The test conditions can be found in Table 2 of the supplement. Protein marker used: PageRuler Prestained Protein Ladder (Thermo Scientific).


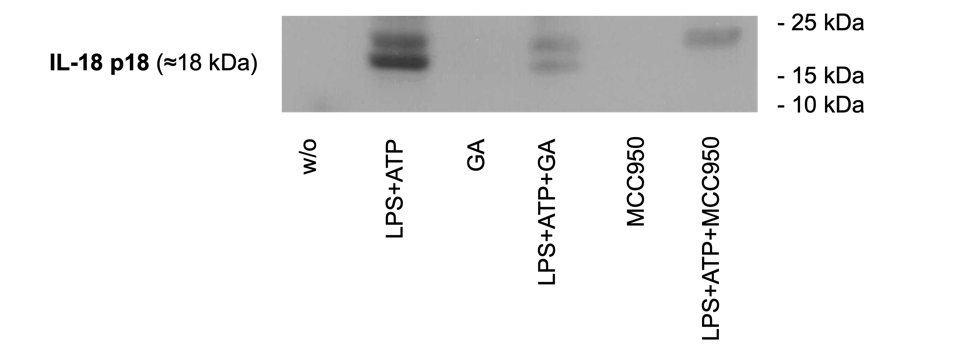


**Supplementary Figure 14**: Unprocessed Western Blot image of IL-18 (supernatant). The test conditions can be found in Table 2 of the supplement. Protein marker used: PageRuler Prestained Protein Ladder (Thermo Scientific).


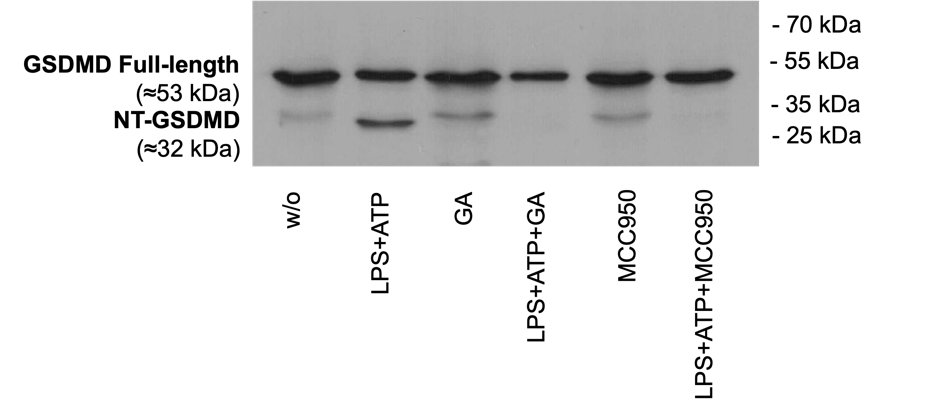


**Supplementary Figure 15**: Unprocessed Western Blot image of GSDMD (cell lysate). The test conditions can be found in Table 2 of the supplement. Protein marker used: PageRuler Prestained Protein Ladder (Thermo Scientific).


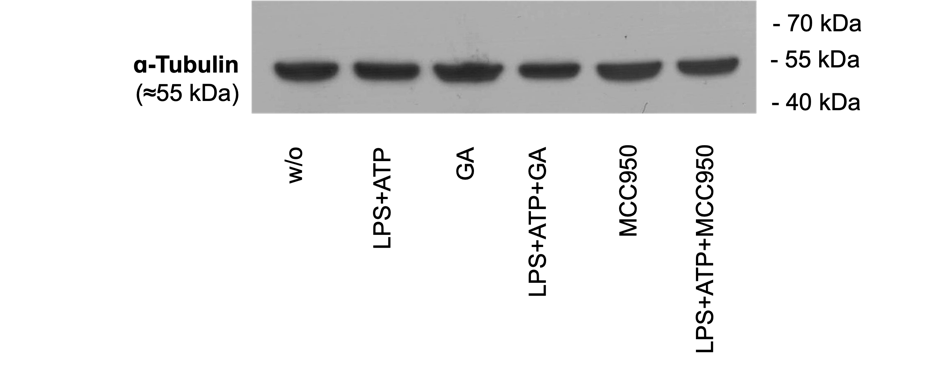


**Supplementary Figure 16**: Unprocessed Western Blot image of α-tubulin (cell lysate). The test conditions can be found in Table 2 of the supplement. Protein marker used: PageRuler Prestained Protein Ladder (Thermo Scientific).


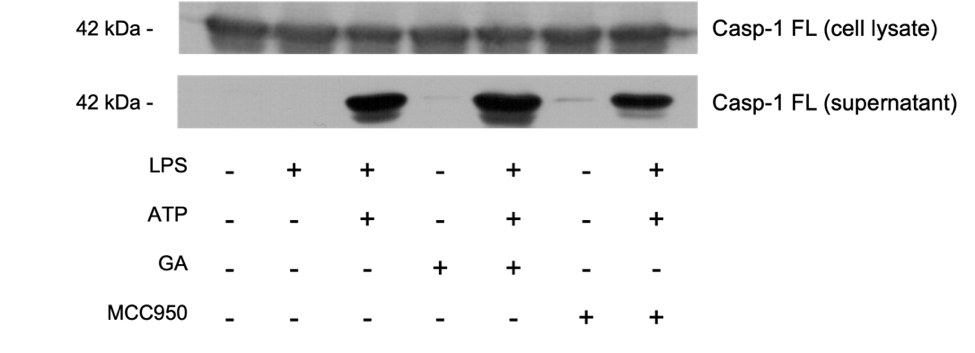


**Supplementary Figure 17**: Western blot image of caspase-1 (FL) in cell lysates and supernatants of J774A.1 macrophages.
